# Adenosine Deaminase Inhibitory Activity of Medicinal Plants: Boost the Production of Cordycepin in *Cordyceps militaris*

**DOI:** 10.3390/antiox12061260

**Published:** 2023-06-12

**Authors:** Ayman Turk, Solip Lee, Sang Won Yeon, Se Hwan Ryu, Yoo Kyong Han, Young Jun Kim, Sung Min Ko, Beom Seok Kim, Bang Yeon Hwang, Ki Yong Lee, Mi Kyeong Lee

**Affiliations:** 1 College of Pharmacy, Chungbuk National University, Cheongju 28160, Republic of Korea; aymanturk@chungbuk.ac.kr (A.T.); dudaos000@chungbuk.ac.kr (S.L.); sangwon1352@chungbuk.ac.kr (S.W.Y.); sehwan0188@chungbuk.ac.kr (S.H.R.); ko-sung-min@hanmail.net (S.M.K.); kbsneo@naver.com (B.S.K.); byhwang@chungbuk.ac.kr (B.Y.H.); 2 College of Pharmacy, Korea University, Sejong 47236, Republic of Korea; yookyong05@korea.ac.kr (Y.K.H.); yjkim22@korea.ac.kr (Y.J.K.); 3 C&G Agricultural Association, Sejong 30067, Republic of Korea

**Keywords:** *Cordyceps militaris*, Mori Folium, adenosine deaminase (ADA), cordycepin, medicinal plants

## Abstract

Cordycepin, also known as 3′-deoxyadenosine, is a major active ingredient of *Cordyceps militaris* with diverse pharmacological effects. Due to its limited supply, many attempts have been conducted to enhance the cordycepin content. As part of this study, eight medicinal plants were supplemented with cultivation substrates of *Cordyceps* to increase the cordycepin content. *Cordyceps* cultivated on brown rice supplemented with Mori Folium, Curcumae Rhizoma, Saururi Herba, and Angelicae Gigantis Radix exhibited increased cordycepin content compared to a brown rice control. Among them, the addition of 25% Mori Folium increased the cordycepin content up to 4 times. Adenosine deaminase (ADA) modulates the deamination of adenosine and deoxyadenosine, and the inhibitors have therapeutic potential with anti-proliferative and anti-inflammatory properties. As ADA is also known to be involved in converting cordycepin to 3′-deoxyinosine, the inhibitory activity of medicinal plants on ADA was measured by spectrophotometric analysis using cordycepin as a substrate. As expected, Mori Folium, Curcumae Rhizoma, Saururi Herba, and Angelicae Gigas Radix strongly inhibited ADA activity. Molecular docking analysis also showed the correlation between ADA and the major components of these medicinal plants. Conclusively, our research suggests a new strategy of using medicinal plants to enhance cordycepin production in *C. militaris*.

## 1. Introduction

Cordycepin is a nucleoside analog which is also known as 3′-deoxyadenosine. It is a major active ingredient of *Cordyceps militaris* with diverse pharmacological effects such as anticancer, anti-viral, and immunostimulatory activity [1,2,3]. The *Cordyceps* mushroom is a parasitic fungus that develops on the bodies of insects in the wild. Therefore, it is also known as “winter worm summer grass”. *Cordyceps* were originally collected in nature, but it is very rare and difficult to find, so efforts are being made to obtain it. Fortunately, *C. militaris* can be cultivated artificially and supplied via cultivation. In addition, various efforts are being made to produce *Cordyceps* with increased cordycepin content, the main active ingredient. Many investigations have focused on improving the cultivation conditions, using methods such as changes in the medium, including substrates and additives [4,5,6,7]. Grains have been utilized extensively as substrates for the cultivation of *Cordyceps* owing to their accessibility and low cost [6]. We recently reported that *Cordyceps* grown in a medium containing insects had a much higher cordycepin content compared to that on grain. In addition, various additives, such as fatty acids, also led to an increase in cordycepin content [6,7].

Adenosine deaminase (ADA), also known as adenosine aminohydrolase, is a zinc-containing metalloenzyme with a molecular weight of 41 kDa, present in plants, bacteria, and humans [8]. It is a key enzyme in purine metabolism, which deaminates adenosine and 2′-deoxyadenosine to inosine and 2′deoxyinosine, respectively. Both adenosine and 2′-deoxyadenosine serve essential roles in the body; ADA plays an important in adenosine homeostasis and nucleoside metabolism [9]. ADA regulates immune responses, therefore, ADA is reported to be related to immune deficiencies such as acquired immunodeficiency syndrome (AIDS) and leukemia. Abnormal activity of ADA also progresses to various diseases such as inflammation and cancer [10,11]. ADA can also deaminate adenosine analogs used in many therapies [12]. 

**Figure 1:**
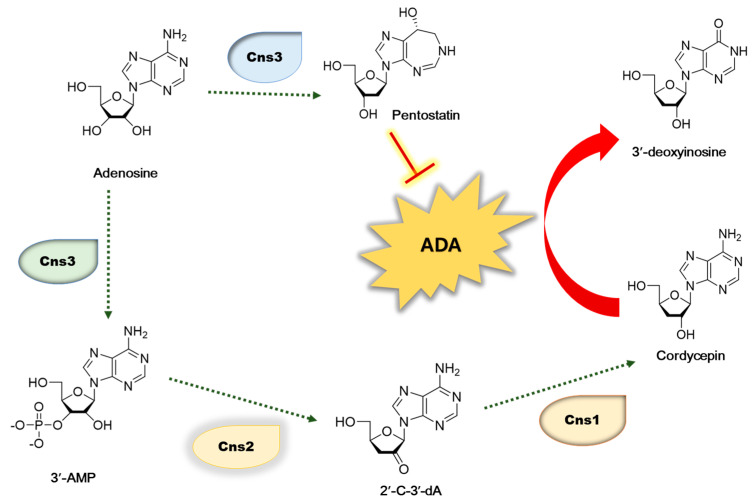
Biosynthetic pathway of cordycepin in C. militaris. 3′-AM—adenosine-3′-monophosphate; 2′-C-3′-Da—2′-carbonyl-3′-deoxyadenosine.

As cordycepin has nutritional and pharmaceutical values, many researchers have extensively investigated its biosynthesis pathway [13,14,15]. Adenosine is phosphorylated to 3′-AMP by Cns3 and further dephosphorylated to 2′-C-3′-dA by Cns2. Then, cordycepin is synthesized from 2′-C-3′-dA by the product of Cns1. Therefore, the cordycepin content is regulated by the transcription levels of cordycepin biosynthetic genes including *cns1*, *cns2*, and *cns3* (Figure 1). Therefore, we investigated the effect of oleic acid, which showed a high correlation with cordycepin content, on the expression of these genes [7]. The content of the cordycepin is also controlled through the degradation of cordycepin by various factors. Among them, ADA is an important factor that degrades cordycepin. Due to its structural resemblance to adenosine, cordycepin is likewise a substrate of ADA. The Cordycepin metabolite 3′-deoxyinosine was discovered, showing that ADA was responsible for converting cordycepin to 3′-deoxyinosine, which reduced the cordycepin content (Figure 1) [16]. It has also been reported that oxidative stress causes the degradation of cordycepin [17,18], and antioxidant extracts significantly reduce the action of ADA [19].

As increased ADA activity is related to many pathological conditions, ADA inhibitors are suggested as important targets for developing anti-proliferative, anti-inflammatory, and immunosuppressive therapeutics. Accordingly, there have been reports of a significant number of ADA inhibitors originating from both naturally occurring and manufactured sources [20,21,22]. There is an ongoing investigation into the development of ADA inhibitors that are both more effective and less toxic, with the goal of their potential application in medical scenarios [23]. Pentostatin, a nonspecific ADA inhibitor has been clinically used for the treatment of leukemia and lymphoma. Phenolic compounds, including flavonoids, are important groups of bioactive compounds responsible for various beneficial effects of plants. Flavonoids such as quercetin, kaempferol, and daidzein are reported to inhibit ADA activity [23,24,25,26]. Related to the regulation of cordycepin content, pentostatin is reported to preserve the cordycepin from breakdown by the inhibition of ADA activity. In addition, several ADA inhibitors are suggested to increase the cordycepin content [23].

We continue our research to obtain *Cordyceps* with increased cordycepin content through changes in cultivation conditions, including substrate composition. Medicinal plants contain various metabolites which are expected to affect *Cordyceps* growth and cordycepin synthesis. In this study, eight medicinal plants including *Camellia sinensis* leaves (Theae Folium), *Curcuma longa* rhizomes (Curcumae Rhizoma), *Linum usitatissiumum* seeds (Lini Semen), *Saururus chinensis* leaves (Saururi Herba), *Dioscorea oppositifolia* roots (Dioscoreae Rhizoma), *Morus alba* leaves (Mori Folium), *Angelica gigas* roots (Angelicae Gigantis Radix), and *Ulmus macrocarpa* roots (Ulmi Cortex) were selected as additives to substrates of *Cordyceps* cultivation. Each medicinal plant investigated in this study contains different types of metabolites. Theae Folium contains caffeine along with phenolic compounds, Curcumae Rhizoma contains diarylhepanoids, and Lini Semen is rich in fatty acids [27,28,29]. Saururi Herba contains flavonoids and alkaloids, Dioscoreae Rhizoma contains steroidal saponins, whereas Mori Folium contains phenolic compounds and flavonoids [30,31,32,33]. Angelicae Gigantis Radix and Ulmi Cortex contain coumarin and triterpenes as their main constituents, respectively [34,35]. Furthermore, different parts of the selected plants were used, such as leaves, roots, cortex and seeds, a comparison of the parts used has also been considered. *Cordyceps* were cultivated in substrates containing the selected medicinal plants, and the effect on their growth was observed, and the content of cordycepin in the *Cordyceps* was measured. 

The inhibitory activity of ADA was further investigated for medicinal plants that increased the cordycepin content of *Cordyceps*. In addition, metabolomic analysis was performed for extracts of medicinal plants and molecular docking analysis was performed for the main components of the medicinal plants to explain the inhibitory effect on ADA. We also measured the antioxidant activity of the extracts of medicinal plants, and we suggest synergistic effects on the cordycepin content.

## 2. Materials and Methods

### 2.1. Raw Materials

Leaves of Camellia sinensis, rhizomes of Curcuma longa, seeds of Linum usitatissiumum, aerial parts of Saururus chinensis, roots of Dioscorea oppositifolia, leaves of Morus alba, roots of Angelica gigas, and cortex of Ulmus macrocarpa were purchased from the local herbal market, Chungbuk, Korea, in October 2020. A voucher specimen of CBNU2020-TH for Theae Folium, CBNU2020-CR for Curcumae Rhizoma, CBNU2020-LS for Lini Semen, CBNU2020-SH for Saururi Herba, CBNU2020-DR for Dioscoreae Rhizoma, CBNU2020-MF for Mori Folium, CBNU2020-AGR for Angelicae Gigantis Radix and CBNU2020-UC for Ulmi Cortex was deposited at the herbarium of the College of Pharmacy, Chungbuk National University, Korea.

### 2.2. Cultivation of C. militaris

The stock culture was maintained on potato–dextrose–agar (PDA) slants with 20.0 g/L glucose, 3.0 g/L KH_2_PO_4_, and 1.5 g/L MgSO_4_·7H_2_O. The seed culture was grown in PDA medium at 25 °C for 13 days after being moved from an active slant to a Petri plate, and then chilled to 4 °C for further subculturing. A sterile cylinder cutter removed a 1 cm swatch of PDA plate culture for use as the inoculum. The seed culture was injected into a 500 mL culture container with a diameter of 8.5 cm and a height of 14.0 cm to start the surface culture. Each polypropylene container was stuffed with eight different MP at two different ratios, 15% and 25%, and then sterilized in an autoclave for 30 min at 121 °C. On a spotless workbench, we brought the polypropylene container to room temperature and added inoculum at a ratio of 1:2 (*v/w*) [7].

### 2.3. Quantification of Cordycepin

Cordycepin was quantitated by HPLC analysis, as previously reported [6]. One gram of ground *Cordyceps* was mixed with 10 mL of 80% methanol and left at room temperature for 24 h. After passing through a 0.45 m PTFE filter, the solvent was taken out of the extract by evaporating it in a vacuum. The dried extract was mixed with methanol up to 10 mg/mL. For a high-pressure liquid chromatography (HPLC) test, the solutions were kept at –20 °C. An HPLC system with Waters 600 Q-pumps, a 996-photodiode array detector, and Waters Empower software was used to measure how much cordycepin there was. The separation was performed on an RP-C18 column (5 μm, 10 mm × 150 mm) with a mixture of methanol and water (12:88, *v/v*) used for isocratic elution. During the whole assay, the amount injected was 10.0 μL, and the flow rate of the solvent was 2 mL/min. All separations were performed at room temperature with a 40 min run time and a detection wavelength of 260 nm.

### 2.4. HPLC-Q-TOF MS

A Shiseido CapCell PAK C18 column (5 μm, 4.6 mm I.D. × 150 mm) with a C18 guard column (4.00 × 3.00 mm; Phenomenex, Torrance, CA, USA) was used for the HPLC analysis. The injection volume was 10 μL, and the flow rate was 0.6 mL/min. The mobile phase consisted of water containing 0.1% formic acid (A) and acetonitrile containing 0.1% formic acid (B). Gradient elution was as follows: 0–5 min, 5% B, 5–30 min, and 5–95% B. The eluted fractions were utilized for mass chromatography analysis after UV detection. The elution fractions were ionized by ESI in negative and positive modes, and parallel alternating scan mode MS and MS/MS spectrometric data acquisition was performed. The mass parameters were as follows: nebulizer pressure, 40 psi; capillary voltage, 4000 V; fragmentor voltage, 175 V; skimmer voltage, 65 V; drying gas temperature, 325 °C; flow rate of drying gas, 12.0 L/min; collision energies, 10, 20, 30, and 40 V; mass scan range, *m*/*z* 50–1700. All acquisition parameters were adjusted using the MassHunter Workstation software LC/MS data acquisition for 6530 series Q-TOF (version B.05.00).

### 2.5. Determination of ADA Inhibitory Activity

The ADA inhibitory activity was assayed based on the reaction mechanism of ADA. The enzyme assay was performed in a 1.0 mL reaction solution at 37 ℃ for 10 min. The reaction solution consisted of 500 µL of enzyme solution, 50 µL of cordycepin solution (10 mg/mL), 50 µL of sample solution (10 mg/mL), and 400 µL of 50 mM phosphate buffer saline (pH 7.4). The ADA inhibitory activity was determined by measuring the degradation of cordycepin in a Waters 515 HPLC pump with a 996-photodiode array detector, and Waters Empower software using a Gemini-NX ODS-column (150 × 21.2 mm). The mobile phase was methanol and water (12:88) at a flow rate of 2.0 mL/min. The effluent was monitored at 260 nm. Quantitative analysis of cordycepin was based on the peak area and its standard curve in HPLC. The ADA inhibitory activity was expressed as the percentage inhibition of cordycepin degradation.

### 2.6. Determination of Antioxidant Activity Using DPPH Assay

The free radical scavenging activity measurement utilizing DPPH was used to determine the antioxidant activity, as reported in the prior study [36]. The absorbance was determined using 550 nm as the wavelength. A positive control was carried out with the application of ascorbic acid. The DPPH reduction was determined by using the formula AA (%) = ((A0−A1)/A0) ×100, in which AA represents the antioxidant activity (percent), A0 denotes the absorbance of the blank sample, and A1 represents the absorbance of the concentration that was being tested.

### 2.7. LC-Q-TOF-MS

The chemical components from the medicinal plants were analyzed using a previously reported technique. Briefly, high-performance liquid chromatography (HPLC) analysis was performed using the Agilent 1260 HPLC series system (Agilent, Santa Clara, CA, USA). Sample extracts were separated using a Kinetex C18 column (150 × 4.6 mm; 5 μm) with a C18 guard column (4.00 × 3.00 mm; Phenomenex, Torrance, CA, USA). Solvent A (0.1% *v/v* formic acid in H_2_O) and solvent B (0.1% *v/v* formic acid in acetonitrile) were selected for the mobile phase. The gradient program was 15% B (0–5 min) and 90% B (5–30 min), flow rate of 0.6 mL/ min, and injection volume of 10.0 μL. The Agilent 6530 Q-TOF mass spectrometer (Agilent) with ESI in the positive and negative modes was used for the mass chromatography analysis.

### 2.8. Molecular Docking Analysis

The three-dimensional (3D) structure of human adenosine deaminase (PDB-ID: 3IAR) was obtained from the Protein Data Bank “http://www.rcsb.org/pdb (accessed on 29 May 2023) and was used in the in silico analysis. Minimization of the protein structure was carried out using force field OPLS4 until the average root-mean-square deviation (RMSD) of the heavy atoms reached 0.3 Å, using the protein preparation tool in Maestro v12.4. The two-dimensional (2D) structures of twelve ligands were drawn by ChemDraw 20.0. The 2D structures of the ligands were converted into 3D structures using the LigPrep tool to obtain the geometry optimized structures at pH 7.0 ± 2.0, with the chirality of the ligand determined by its 3D structure. The final step of LigPrep was an energy minimization of the 3D conformers using OPLS4. The docking grid of the receptor’s active site was detected using the PDB file of the coordinates with the receptor grid generation tool in Maestro v12.4. This site defines the area around the active site in term of coordinates x, y, and z. The receptor grid box resolution was centered at coordinates 7.25, −3.05, and −0.35, corresponding to the x, y and z-axes, respectively. Docking and calculations were run in the standard precision (SP) mode of Glide.

### 2.9. Statistical Analysis

Statistics were reported using means and standard deviations. For statistical analysis, the Statistical Analysis System program was used. An all-phase analysis of variance was employed to examine the differences. Significant differences between means were determined using Duncan’s multiple range tests at a significance level of *p* < 0.05.

## 3. Results and Discussion

### 3.1. Effect of Various Medicinal Plants on Mycelium Growth and Cordycepin Production

*Cordyceps* originally grew on insects in nature. However, due to such a limited quantity that can be obtained in nature, various methods have been attempted to obtain it. Among them, cultivation is considered as an essential alternative strategy to ensure its availability. Cultivation can produce *Cordyceps* mushrooms for cordycepin by adjusting the cultivation conditions, without being affected by the climate or other environmental factors, in a shorter time than growing in nature. In addition, high quality mushrooms can be efficiently produced by the regulation of cultivation conditions such as medium compositions and temperature. Recently, grains have been commonly used for the cultivation of *Cordyceps*. For its practicality and cost-effectiveness, brown rice has been employed as a substrate for *Cordyceps* cultivation in various industries [6]. In addition, optimization of the cultivation conditions was also utilized to obtain excellent *Cordyceps* with a high content of cordycepin, a major bioactive compound [6,7].

Medicinal plants contain various metabolites, which exert diverse effects. Based on our preliminary research on *Cordyceps* cultivation, we supposed that these different types of components in medicinal plants are expected to affect *Cordyceps* growth and cordycepin synthesis. Therefore, eight medicinal plants with different types of metabolites were chosen as substrates for cordycepin cultivation: Theae Folium, Curcumae Rhizoma, Lini Semen, Saururi Herba, Dioscoreae Rhizoma, Mori Folium, Angelicae Gigantis Radix, and Ulmi Cortex. Each dried medicinal plant was added to brown rice with 15% and 25% ratios. After the cultivation of *Cordyceps* using different substrates, the cordycepin content was quantitated by HPLC analysis. 

**Figure 2:**
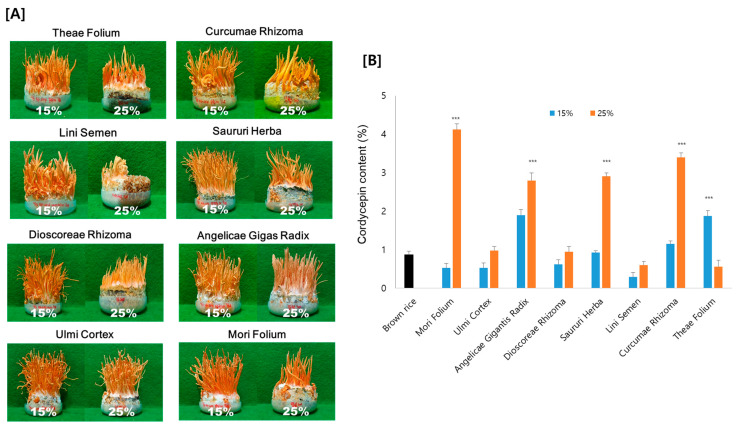
(**A**) Morphology and (**B**) the amount of cordycepin in Cordyceps cultivated on eight different medicinal plants. Cordyceps were cultivated for 35 days at 20 °C, and the cordycepin amount was determined by HPLC analysis. *** *p* < 0.001 vs. Brown rice control (n = 3).

As shown in Figure 2A, Cordyceps developed on all eight of the tested medicinal plant substrates, but the fruiting bodies developed substantially differently for each medicinal plant. The growth of the fruiting bodies also differed depending on the ratio of the medicinal plant in the substrate. Overall, fruiting bodies were better induced at a ratio of 15% compared to 25% medicinal plants. However, the cordycepin content displayed quite different variations compared to the morphology of fruiting bodies. Considering the ratio of medicinal plants, seven samples grown with 25% medicinal plants indicated a greater cordycepin content than those grown with 15%. As an exception, with Theae Folium, the content of cordycepin increased when 15% of Theae Folium was added, but the cordycepin content was almost unchanged compared to the brown rice control with 25% Theae Folium addition. Among the medicinal plants added, Cordyceps grown on Mori Folium exhibited the greatest cordycepin concentration, 42.3 mg cordycepin per g dried Cordyceps, followed by Curcumae Rhizoma, Saururi Herba, and Angelicae Gigantis Radix (Figure 2B). 

These results support the relevance of medicinal plants for cordycepin synthesis. It might be possible to produce Cordyceps with an increased cordycepin content by using medicinal plants as substrates. Cordyceps grown on 25% Mori Folium contained 4 times more cordycepin than those grown on brown rice as a control. Since the growth of fruiting bodies and the production of cordycepin appeared differently depending on the type of plant and the amount added, optimization is necessary for the application of the technique.

### 3.2. Effects of Medicinal Plants on ADA Inhibition

Since the addition of medicinal plants to the substrate of Cordyceps cultivation induced an increase in cordycepin content, we tried to investigate the mechanism. As described above, ADA was responsible for converting cordycepin to 3′-deoxyinosine, which reduced the content of cordycepin [13]. Pentostatin, a nonspecific ADA inhibitor, has been demonstrated to preserve cordycepin from its breakdown, and further boost the effectiveness of cordycepin treatment [37]. Research on the ADA inhibitory effect of medicinal plants is being conducted, and various compounds have been reported to inhibit ADA activity efficiently [38,39]. Therefore, we tried to confirm whether the increases in cordycepin by the medicinal plants are mediated by the inhibition of ADA activity. 

**Figure 3:**
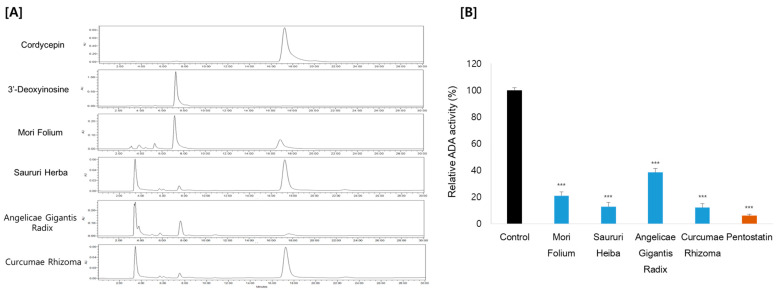
(**A**) HPLC chromatograms and (**B**) ADA activity of medicinal plants. Pentostatin was used as a positive control. *** *p* < 0.001 compared to the control.

**Figure 4:**
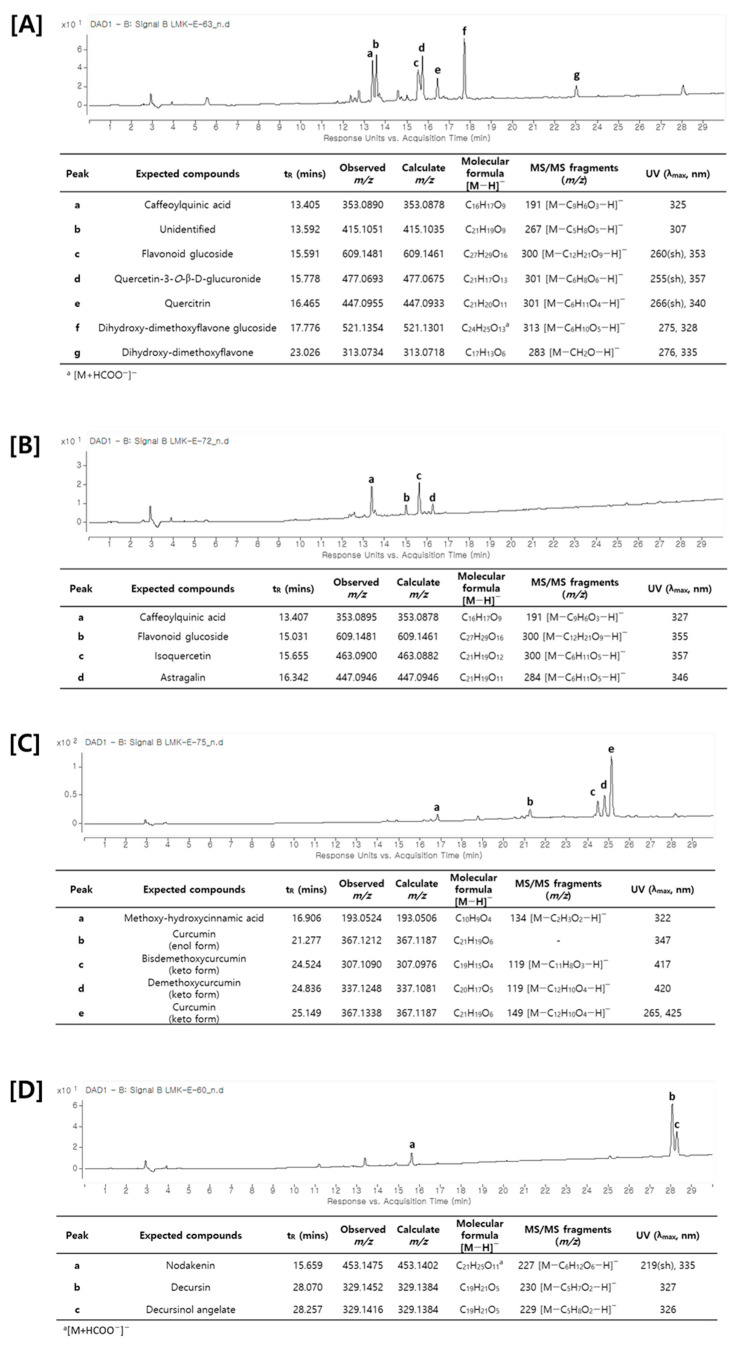
UV chromatogram and identification of major compounds by LC–MS/MS analysis. (**A**) Saururi Herba, (**B**) Mori Folium, (**C**) Curcumae Rhizoma, and (**D**) Angelicae Gigantis Radix.

HPLC with ultraviolet spectroscopy was employed to measure putative ADA inhibitory effects using cordycepin as the substrate. This method involves monitoring the drop in the substrate concentration and the rise in the product 3′-deoxyinosine by the action of ADA. Consistent with the cordycepin content, Curcumae Rhizoma and Sauri Herba remarkably decreased the ADA activity, by 88% and 87%, respectively. Mori Folium and Angelicae Gigantis Radix also showed ADA inhibition, by 79% and 62%, respectively (Figure 3). These results suggest that the increase in cordycepin content in Cordyceps cultivated in the medium supplemented with these medicinal plants is probably exerted by the inhibitory effect on ADA. In order to characterize the ADA inhibitory components of these medicinal plants, metabolite profiles were analyzed by LC–MS/MS analysis (Figure 4). We further examined the ADA inhibitory efficacy of the main components using molecular docking analysis. 

A metabolite analysis using LC–MS/MS was attempted to analyze the main components of the plants that increased the production of cordycepin. As shown in Figure 4, an HPLC chromatogram of each medicinal plant was obtained and the main components were identified through LC–MS/MS analysis. As described above, the main components of each medicinal plant were shown to have different types of chemical structures. Saururi Herba and Mori Folium mainly contain phenolic compounds and flavonoids such as caffeoylquinic acid, quercetin-3-*O*-β-d-glucuronide, quercitrin, isoquercetin, and astragalin. Curcumae Rhizoma contains diarylheptanoid derivatives such as curcurmin, demethoxycurcumin, and bisdemethoxycurcumin, whereas Angelica Gigantis Raeix contains coumarins such as decursin and decursinol angelate. Based on these data, the ADA inhibitory efficacy of these main components was predicted using molecular docking.

**Figure 5:**
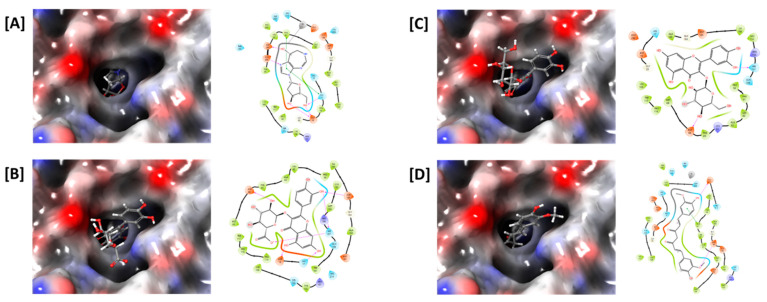
In silico docking study of ADA with (**A**) pentostatin, (**B**) quercetin-3-O-β-d-glucuronide, (**C**) isoquercetin, and (**D**) curcumin. The ligand-binding pocket of ADA is shown as a transparent surface (left panels). Interactions between amino acid residues of ADA and ligands, such as hydrogen bonds (pink) and pi-pi stacking interactions (green), are shown (right panels).

Similar to the ADA inhibitory effects of the medicinal plant extracts, the major components also showed high docking scores against the ADA enzyme (Table 1). The ADA inhibitory efficacy differed depending on the chemical structures of the compounds. Among the constituents, quercetin-3-*O*-β-d-glucuronide of Saururi Herba showed the highest docking score (−7.292), followed by isoquercetin of Mori Folium (−6.676) and curcumin of Curcumae Rhizoma (−6.247). Decursin and decursinol angelate, the major components of Angelicae Gigantis Radix, showed relatively low docking scores of −5.147 and −4.642, respectively, in our analysis. Interactions between ADA and the compounds, such as hydrogen bonds and pi-pi stacking interactions, were also observed (Figure 5), supporting the ADA inhibitory potentials of these compounds. Collectively, these data suggest that the cordycepin content was increased by the addition of medicinal plants containing these ADA inhibitory compounds.

### 3.3. Cordycepin Production by the Regulation of ADA 

Cordycepin, a major bioactive compound of *C. militaris*, has important nutritional and pharmaceutical values with various effects. Therefore, obtaining cordycepin to use and develop efficiently is required. Many factors have been reported to regulate the content of cordycepin. Cordycepin is known to be synthesized from adenosine by the cordycepin synthetic genes such as Cns1, Cns2, and Cns3. The content of cordycepin is also regulated by the degradation process, mainly mediated by ADA. In addition, oxidative stress has been reported to affect the cordycepin content by the control of its degradation. Due to the importance of cordycepin, many efforts have been made to increase the cordycepin content in *Cordyceps*. We previously reported that oleic acid increased cordycepin biosynthesis by the enhancement of *cns1* and *cns2* transcriptions [7]. 

Here, we investigated the effect of ADA inhibition on cordycepin content. *Cordyceps* were cultivated by substrates supplemented with several kinds of medicinal plants and the changes in cordycepin content were measured. The ADA inhibitory efficacy of the medicinal plants which induced the increased cordycepin content was also measured. The medicinal plants with a stimulatory effect on the cordycepin content significantly inhibited the ADA activity as measured by the UV-HPLC method using cordycepin as the substrate. To investigate the mechanism of these medicinal plants on the cordycepin increase and ADA inhibition, metabolites of each medicinal plant were analyzed using LC–MS/MS analysis. Further, molecular docking analysis of the main components of these medicinal plants showed high scores against ADA. The binding between ADA enzyme and compounds also explained the inhibitory potential of the compounds. The inhibitory activity on ADA is quite different depending on the chemical structures of compounds. Interestingly, caffeoylquinic acid, an ADA inhibitory compound, is present in both Saururi Herba and Mori Folium. Therefore, it is thought that the ADA inhibitory effect of the extract is affected by the structures of the constituents and the content of each compound in the medicinal plants. The synergistic effects of several compounds in each plant might also affect the efficacy. Saururi Herba contains not only caffeoylquinic acid but also quercetin-3-*O*-β-d-glucuronide with a high docking score, which contributed to the higher effectiveness. In addition to ADA inhibition, several factors are known to affect the growth of *Cordyceps* and the stabilization of cordycepin. Oxidative stress is one of the factors involved in the inhibition of *Cordyceps* growth and the degradation of cordycepin. Therefore, the antioxidant potentials of medicinal plants were measured using the DPPH radical scavenging assay. All four medicinal plants showed strong antioxidant activities, consistent with previous studies [40,41]. Mori Folium and Curcumae Rhizoma showed strong DPPH radical scavenging activities of 94.27 ± 3.4% and 94.27 ± 4.8%, respectively, at concentrations of 100 μg/mL. Angelicae Gigantis Radix and Saururi Herba also showed excellent radical scavenging activities of 82.83 ± 4.2% and 78.87 ± 6.5%, respectively. Thus, the antioxidant potentials of medicinal plants might contribute to the stabilization of cordycepin. Conclusively, our research will provide some strategies for producing cordycepin by ADA inhibition and antioxidant potential using medicinal plants.

## 4. Conclusions

This study showed the possibility of using medicinal plants as substrates for growing *Cordyceps* with enhanced cordycepin production. Cordycepin yields were increased by addition of medicinal plants such as Mori Folium, Saururi Herba, Curcurmae Rhizoma, and Angelicae Gigantis Radix to the substrates for *Cordyceps* cultivation. The cordycepin content was increased by as much as 4 times by the addition of 25% Mori Folium to the brown rice substrate. These medicinal plants remarkably inhibited the ADA activity, which regulates the conversion of cordycepin to 3ʹ-deoxyinosine. Further, molecular docking analysis demonstrated the inhibitory effect of the main compounds of these medicinal plants against ADA and showed binding potential between ADA and the active compounds. Therefore, the present study suggested the inhibitory activity of medicinal plants on ADA and thus on cordycepin production. It is hoped that the results of this study can be used to obtain excellent *Cordyceps* with increased cordycepin content.

## Figures and Tables

**Table 1:** Docking score of the ligands with the active site of adenosine deaminase (PDB-ID: 3IAR).

Origin	Compounds	Docking Score
Positive control	Pentostatin	−7.738
Saururi Herba	Caffeoylquinic acid	−5.740
	Quercetin-3-*O*-β-d-glucuronide	−7.292
	Quercitrin	−4.377
Mori Folium	Caffeoylquinic acid	−5.740
	Isoquercetin	−6.676
	Astragalin	−4.903
Curcumae Rhizoma	Bisdemethoxycurcumin	−5.598
	Demethoxycurcumin	−5.915
	Curcumin	−6.247
Angelicae Gigantis Radix	Decursin	−5.147
	Decursinol angelate	−4.642

## Data Availability

The data presented in this study are available on request from the corresponding author.
